# Alarming Result of Tight-Lacing

**Published:** 1887-07-30

**Authors:** 


### ALARMING RESULT OF TIGHT-LACING.

(*Scotch Paper.*)

Not a sound was heard, not a ghost of a note,
Though she long at the music was seated:
But a whisper passed round in the corners remote,
As she fled from the atmosphere heated.

She thought as she rushed to the door of the room,
And, fainting, in vain tried to force it,
That death before morning would be her sad doom,
So fatally tight was her corset.

They carried her gently and placed her in bed,
And ripped up the death-gripping "body";
They sprinkled pure eau-de-cologne on her head,
And gave her some hot whiskey-toddy.

She said, as she opened her violet eyes,
The scene in the bedroom embracing,
"Never more I the doctor's advice will despise,
Never more try that horrid tight-lacing."

